# Neuro-Inflammatory Response and Brain-Peripheral Crosstalk in Sepsis and Stroke

**DOI:** 10.3389/fimmu.2022.834649

**Published:** 2022-04-07

**Authors:** Lena Bourhy, Aurélien Mazeraud, Fernando A. Bozza, Guillaume Turc, Pierre-Marie Lledo, Tarek Sharshar

**Affiliations:** ^1^ Institut Pasteur, Université de Paris, Centre National de Recherche Scientifique, Unité Mixte de Recherche (CNRS UMR) 3571, Perception and Memory Unit, Paris, France; ^2^ Neuro-Anesthesiology and Intensive Care Medicine, Groupe Hospitalier Universitaire (GHU) Paris Psychiatrie et Neurosciences, Université de Paris, Paris, France; ^3^ National Institute of Infectious Disease Evandro Chagas (INI), Oswaldo Cruz Foundation (FIOCRUZ), Rio de Janeiro, Brazil; ^4^ D’Or Institute for Research and Education (IDOR), Rio de Janeiro, Brazil; ^5^ Department of Neurology, GHU Paris Psychiatrie et Neurosciences, Université de Paris, Paris, France

**Keywords:** immune response, ischemic stroke, neuromodulation, sepsis, sickness behavior

## Abstract

Despite recent therapeutic advances, ischemic stroke is still a leading cause of death and disability. There is renewed attention on peripheral inflammatory signaling as a way of modulating the post-ischemic neuro-inflammatory process. The immune-brain crosstalk has long been the focus for understanding the mechanisms of sickness behavior, which is an adaptive autonomic, neuroendocrine, and behavioral response to a peripheral inflammation. It is mediated by humoral and neural pathways that mainly involve the circumventricular organs and vagal nerve, respectively. In this review we address the question of how sepsis and stroke can dysregulate this adaptive response, notably by impairing the central integration of peripheral signaling, but also by efferent control of the immune response. We highlight the potential role of gut–brain and brain–spleen signaling in stroke.

## Introduction

Ischemic stroke is a leading cause of death and disability worldwide, with major consequences at personal, social, and economic levels (1). There have been therapeutic advances in reducing acute ischemic injury, notably through recanalization strategies using intravenous thrombolysis (2) and mechanical thrombectomy (3). However, there is an urgent need for treatments that would hamper the ischemia-mediated neurotoxic processes and foster repair and plasticity (4). This strategy should be based on a comprehensive understanding of ischemic stroke pathophysiology. There is currently renewed focus on the ischemia-induced neuro-inflammatory process, notably for its modulation by inflammatory signaling proceeding from the periphery (5). This immune-brain crosstalk has been the subject of study in the psychoneuroimmunology field for some time, especially as it relates to the mechanisms of sickness behavior (6).

Sickness behavior is a physiological integrative reaction to a systemic inflammatory response, particularly induced by infection, which involves the interconnected autonomic, neuroendocrine, and limbic systems. It is stereotypically characterized by social withdrawal, decreased cognition, psychomotor slowing, attention disorders, altered alertness (insomnia, hypersomnia, fatigue, somnolence), mood disorders (irritability, anxiety, depression) and eating behavior changes (anorexia, weight loss, thirst) (6, 7). It is considered an adaptive response for protecting the individual from an aggression by modulating *in fine* both the local and the systemic inflammatory response. The brain centers involved in sickness behavior can be activated by two routes, i.e., the humoral and neural pathways, and the behavior is therefore a result of a complex control loop of the inflammatory response. It can be altered at various levels and by structural or functional mechanisms. For instance, alterations in the perception or the integration of peripheral inflammatory signals at the brain level can induce the dysregulation of the neuro-immune feedback. Besides controlling an acute illness, it is also well-established that sickness behavior can be complicated by long-term psychological disorders, notably depression.

## The Physiology of the Neuro-Immune Crosstalk

### The Neural Pathway

Inflammatory mediators released at the site of inflammation can stimulate peripheral nerves as they express specific receptors both to various cytokines and to Damage-Associated Molecular Patterns (DAMPs) and Pathogen-Associated Molecular Patterns [PAMPs (8)]. Indeed, cytokine receptors (TNF, IL-1ß, IL-6, etc.), or Toll-Like receptors (TLRs) have been identified on the membrane of sensory neurons (9–12). DAMPs and toxins, such as α-hemolysin released by *Staphylococcus aureus*, can bind to the peptide formyl receptor 1 or to the ion channels of the peripheral neurons (13), which in turn stimulate the release of neuropeptides. Various channels involved in nociception, such as the voltage-gated sodium channels Nav1.7, Nav1.8 or Nav1.9, or transient receptor potential channels, are also expressed on the surface of peripheral neurons. Their activation by nociceptive stimuli generated at the site of infection results in the firing of an action potential and in the lowering of the nociceptor threshold. In addition to the sensing role of sensory neurons, afferent neurons modulate the local immune response through the release of neuropeptides (substance P, calcitonin gene-related peptide, vasoactive intestinal peptide, etc.), which interact with endothelium and immune cells located in the vicinity of the axon terminals (8, 14, 15). The vagal nerve has been the most studied of the peripheral nerves involved in immune-brain crosstalk. The vagal afferents, whose cell bodies are in the nodose ganglia, have visceral and thoracic afferents that project to the nucleus of the tractus solitarius (NTS) and the area postrema (AP), which are both located in the medulla and constitute the so-called vagal complex (16). The vagal nerve also contains motor efferences, initiated by cholinergic neurons of the dorsal vagal motor nucleus. The sensory and motor efferences account for 80 and 20% of the vagal fibers, respectively. It has been shown that the administration of lipopolysaccharide (LPS), cytokines (namely, IL-1β and TNF), or pathogens such as *Campylobacter jejuni*, stimulates the vagal afferent signaling in rodents, as evidenced by the increase in the expression of the neuronal activation marker cFos in the NTS (17–21). In addition, a subdiaphragmatic vagotomy (section of the abdominal branch of the vagal nerve) blocks the occurrence of sickness behavior normally induced by the intraperitoneal administration of LPS (22). Finally, electrophysiological recordings have shown that intraperitoneal administration of TNF or IL-1β induces an increase in vagal nerve activity in mice, which is not observed in TNF and IL-1β receptor knock-out mice (18).

### Humoral Pathway

The humoral pathway involves various structures or cell interactions.

#### Circumventricular Organs

Instead of a blood–brain barrier, the circumventricular organs (CVO) have fenestrated capillaries that allow direct passage of molecules between the general circulation and the brain (6, 7). CVOs are located around the third and fourth ventricles. A distinction is made between secretory CVOs (epiphysis, pituitary, median eminence, etc.) and sensory CVOs, namely, the AP, the subfornical organ, and the vascular organ of the terminal lamina. The latter are access points to the brain for circulating cytokines and chemokines, and also DAMPs and PAMPs, which activate receptors on the surface of endothelial cells and resident microglia and thus induce activation cascades that lead to the local production of IL-1ß, interferon-γ (INF-γ), TNF, and prostaglandin E2 (PGE2). These inflammatory mediators can then spread by simple diffusion in the brain parenchyma and bind to neuronal receptors. They also allow the migration of circulating immune cells into the brain. Indeed, studies in mouse models of peripheral inflammation or autoimmune encephalitis have demonstrated the presence of non-microglial leukocytes in some CVOs (7, 23, 24). In addition to CVOs, the choroid plexus and the dura also allow the trafficking of immune cells between the peripheral circulation and the cerebrospinal fluid (CSF), or between the CSF and the lymphatic vessels draining the brain, respectively (12, 25–27).

#### Cerebral Endothelial Cells

Cerebral endothelial cells (CECs) also express cytokine receptors, notably for TNFα and IL-1β. The activation of these receptors promotes the induction of the NF-κB signaling pathway, which leads to the production and release of secondary messengers from these endothelial cells. These secondary messengers include nitric oxide (NO) and prostaglandins (PGs) (7, 28). NO acts mainly as a vasodilator and immunomodulator (29). At the same time, the PG-E2 subtype can diffuse due to its lipid nature, and it thus modulates some central brain effects of the systemic inflammatory response. Indeed, PG receptors are located in brain centers involved in sickness behavior (e.g., hypothalamus and amygdala) (30). The activated CECs also release chemokines, which diffuse and interact with the surrounding neuronal and glial cells (31). Similarly, the IL-6 receptor (IL-6R), synthesized on the surface of leukocytes, can detach from the plasma membrane when the leukocyte is activated. IL-6s bind either to the membrane or to the soluble form of IL-6R. In turn, the IL6–IL-6R complex can interact with endothelial cells, which will express, for instance, adhesion molecules such as ICAM-1 (32). Finally, the CEC-circulating leukocyte interaction is another pathway. By using intravital microscopy, it has been shown that CEC–monocyte interactions are increased 5 days after the induction of liver inflammation. This interaction is mediated by the adhesion protein P-selectin and has been shown to be necessary for local microglial activation (33). Similarly, CEC–leukocyte interactions modulate neuronal excitability, as shown in an experimental model of epilepsy (34).

These humoral pathways relay peripheral inflammation to the brain by involving resident glial cells (microglia and astrocytes) and peripheral cells infiltrated into the parenchyma. This therefore results in the *in-situ* release of cytokines and chemokines, but also of second messengers, such as NO or PGs. These mediators maintain and relay the inflammatory signaling to the neurons. It is important to note that the neural and humoral pathways described above have very different temporal patterns of activation, with afferent neural signaling being much faster than the humoral pathway.

### The Central Integration of Neural and Humoral Signaling

**Figure 1:**
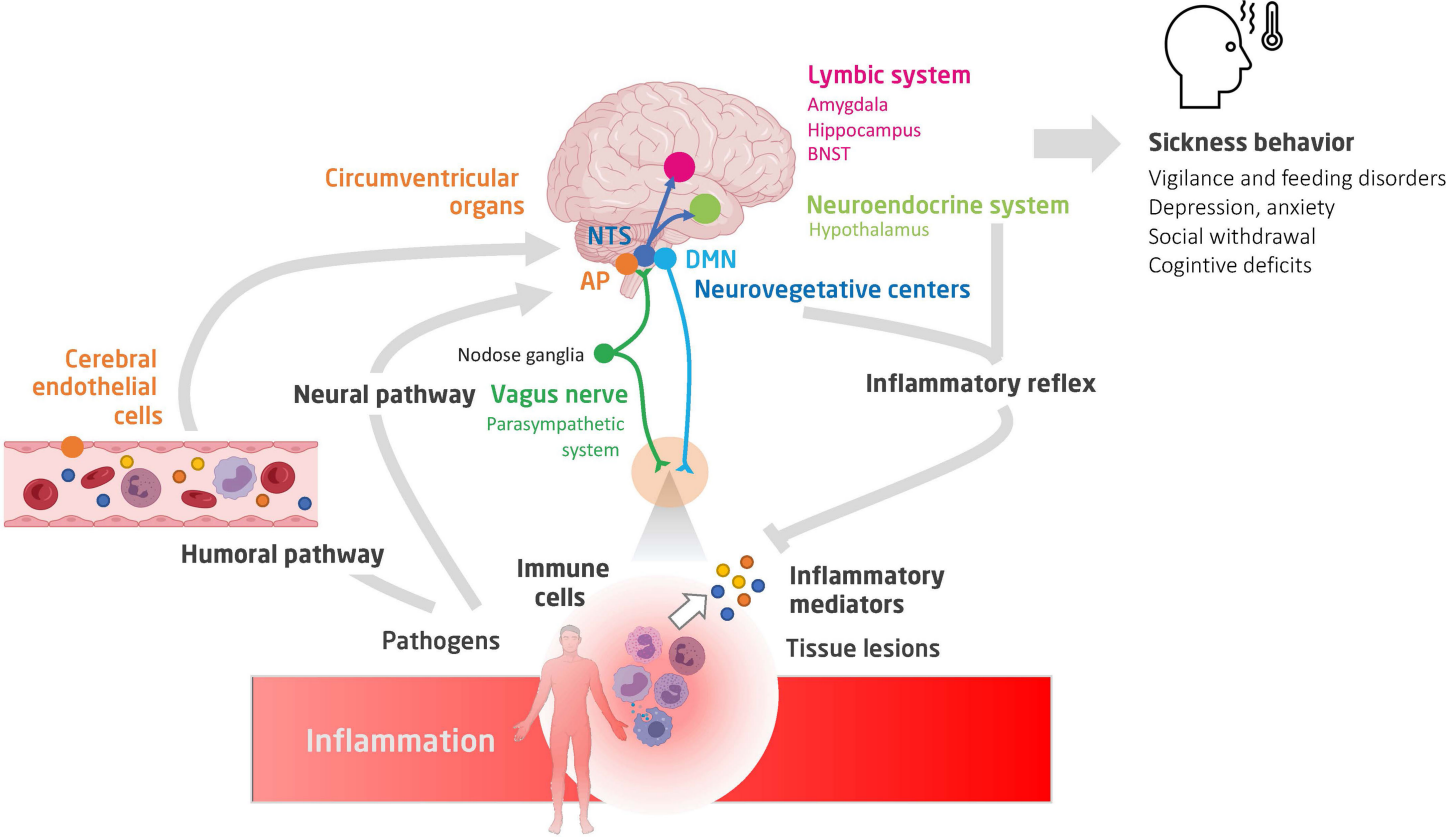
A schematic view of the neuro-immune crosstalk during inflammation. Shown are the humoral and neural pathways conveying inflammatory signaling to the brain, the main brain centers involved in the integration of the inflammatory signaling and controlling autonomic, neuroendocrine and behavioural responses (constituting sickness behavior), and finally, the interaction between peripheral immune cells (notably of the spleen) and stress hormones and autonomic efferences (35, 36). NTS, nucleus of tractus solitarius; AP, area postrema; DMN, dorsal nucleus of vagus nerve; BNST, bed nucleus of the stria terminalis.

The site of action of cytokines and chemokines depends primarily on the areas of expression of their receptors. IL-1β, IFN-γ, and TNF are the cytokines mainly involved in sickness behavior. These receptors are expressed in most resident cell types of the brain and in various brain regions, namely, the CVO, thalamus, striatum, hippocampus, hypothalamus, and amygdala. By studying the expression of early induced-neuronal genes, such as the cFos gene, it is possible to obtain a map of the brain areas that are activated during acute inflammation (**Figure 1**). The vagal complex, which includes the NTS and AP, is activated by both neural and humoral pathways and is the principal entry point for peripheral inflammatory signaling. The vagal complex then transmits the signal to other brainstem nuclei, namely: 1) the rostral ventromedial medullary area (RVLM) that regulates the heart rate, blood pressure and baroreflex; 2) the periaqueductal gray matter (PAG), involved in nociception and defense behavior; 3) the parabrachial nucleus (PBN), that modulates appetitive and aversive responses; and 4) the locus coeruleus (LC), the main center of the sympathetic response to stress (6). In turn, the PBN and LC spread extensively into: 1) the regions of the thalamus that regulate pain perception [paraventricular nucleus (PVT)]; 2) the hypothalamus, which controls the release of stress hormones [paraventricular nucleus (PVN), supraoptic nucleus (SO)], food intake [arcuate nucleus (Arc)], thermoregulation and sleep; and 3) the limbic system, responsible for the sleep cycle (pre-optic median nucleus) and control of cognitive functions and behavioral response [hippocampus, amygdala and nucleus of the bed of the terminal stria (BNST)].

### The Autonomic, Neuroendocrine, and Behavioral Responses

**Figure 2:**
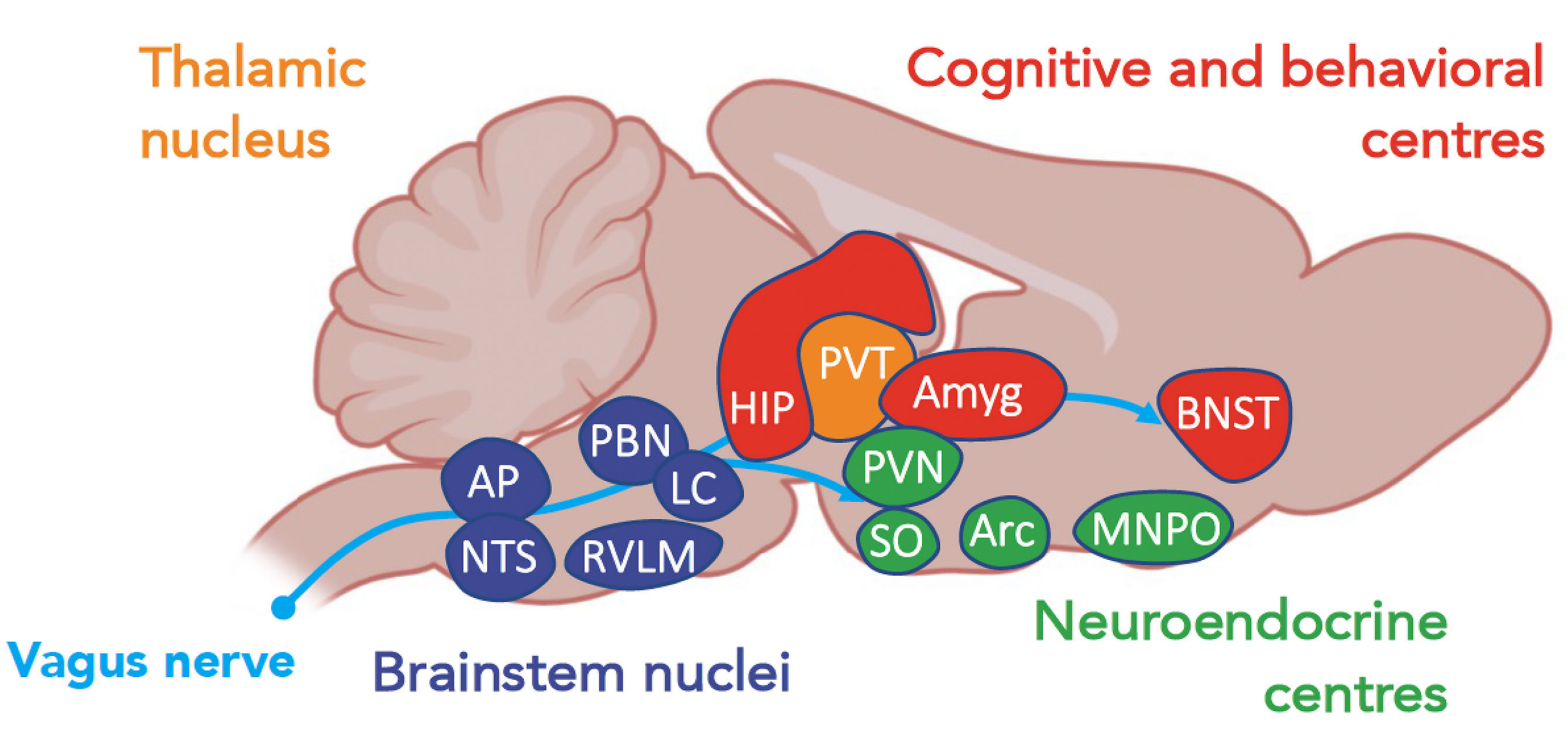
A schematic view of centers, according to their functions, activated during a systemic inflammatory response (37). Amyg, amygdala; AP, area postrema; Arc, arcuate nucleus; BNST, bed nucleus of the stria terminalis; HIP, hippocampus; LC, locus coeruleus; MNPO, median preoptic nucleus; NTS, nucleus of tractus solitarius; PBN, parabrachial nucleus; PAG, periacqueductal gray; PVN, paraventricular nuclei; PVT, paraventricular nuclei of the thalamus; RVLM, rostral ventrolateral medulla; SO, supraoptic nucleus.

The neural and/or humoral-activated brain centers are thus interconnected and orchestrate regulation of the immune response through the so-called ‘inflammatory reflex’ (**Figure 2**).

#### Neural Modulation

Studies on the inflammatory reflex have shown a functional interaction between the parasympathetic (vagal nerve) and sympathetic nervous systems that refine regulation of the innate immune response (8, 38). In this reflex, the inflammatory signal delivered by sensory neurons of the vagal nerve is integrated into the brain. It then stimulates a descending anti-inflammatory response mediated by the cholinergic efferences originating from the dorsal motor nucleus of the vagal nerve (39). Interestingly, these vagal fibers innervate the celiac and superior mesenteric ganglia, which contain noradrenergic neurons of the splenic nerve (40). Therefore, the splenic nerve participates in this inflammatory reflex (41). Indeed, its noradrenergic axons stimulate the splenic T cells, which express both β-adrenergic receptors and the choline acetyltransferase enzyme, which is responsible for acetylcholine (Ach) synthesis (35, 42). Thus, the vagal stimulation of the splenic nerve results in noradrenergic-mediated Ach synthesis and release by spleen T cells. The stimulation of cholinergic neurons controls production of TNF-α in the spleen *via* the splenic nerve (43). In parallel, the neurons of the sympathetic system, located in the spinal cord, innervate many visceral organs (40) and also control the secretion of adrenaline from the adrenal glands, which acts directly as a hormone.

Finally, the immune cells express Ach and/or adrenergic receptors, which modulate pro-inflammatory (i.e., NF-kB) or anti-inflammatory (i.e., JAK) intracellular signaling. For instance, macrophages express the α7 nicotinic acetylcholine receptor (α7nAChR). By binding to β2-adrenergic, the noradrenaline and adrenaline inhibit the NF-κB pathway, and thereby the release of pro-inflammatory cytokines by innate and adaptive immune cells (8). On the other hand, innate immune cells express α-adrenergic receptors that are rather pro-inflammatory (44). Thus, the final immune response depends on various time-dependent factors, namely, cell type, their receptors and environment, and the phase of the inflammatory process.

#### Neuroendocrine Response

The neuroendocrine response to stress involves the hypothalamus–pituitary–adrenal (HPA) axis. Pro-inflammatory cytokines stimulate the release of glucocorticoids by regulating the release of corticotropin hormone by the PVN, of adrenocorticotropic hormone (ACTH) by the ante-hypophysis, and finally of cortisol by the adrenal glands. In turn, glucocorticoids exert a negative control over the immune system by inhibiting the synthesis and release of pro-inflammatory cytokines. In addition, glucocorticoids regulate their own production by negative feedback to higher levels of the HPA (45). A subpopulation of catecholaminergic neurons in the NTS and RVLM projects to the PVN, promoting a corticosteroid response to peripheral inflammation directly mediated by the vagal nerve (46). Vasopressin is also a stress hormone that controls not only blood pressure and the baroreflex but also the HPA axis and the emotional response (47).

#### Behavioral Response

It is striking that some of the late features of infection-induced sickness behavior are comparable to the clinical symptoms of depression. They include altered mood and abilities, reduced sensitivity to reward seeking, and reduced food consumption (37). In humans, the association between depression, anxiety and inflammation has been confirmed by several meta-analyses (37, 48). In the context of systemic inflammation, several brain regions are recruited, including those orchestrating defensive behaviors to ensure survival, notably fear (**Figure 1**). Fear is a rapid adaptive response, which depends on a distributed circuit centered on the amygdala and converging on the central nucleus of the amygdala (CeA). Studies in humans and rodents show that the amygdala is a key relay for fear and anxiety circuits and is directly involved in the behavioral changes observed during illness behavior (49).

## A Dysregulated Response to Stress: The Example of Sepsis

As described previously, sickness behavior is a physiological reaction to an acute systemic inflammatory response that involves interconnected peripheral and central circuits that 1) sense, mediate, and integrate an inflammatory signal, and 2) elaborate an autonomic, neuroendocrine, and behavioral response, leading to immune response modulation and allostasis maintenance. In various conditions, this complex brain-immune crosstalk can dysfunction at any of its relays, resulting in an altered response to stress, either by excess or default. Sepsis represents the model of dysregulated response to stress (50). Thus, septic patients can develop relative adrenal insufficiency. This is associated with increased mortality, which can, however, be reduced by a substitutive opotherapy (51, 52). Impaired osmoregulation of vasopressin also occurs in the acute phase of severe sepsis, resulting from a depolarization of osmoreceptors (53–55). Interestingly, osmoreceptors are located within CVOs and their dysfunction might be induced by inflammatory cytokines. The defective osmoregulation can persist after recovery from sepsis and accounts for the impairment of thirst (53). Furthermore, severe sepsis can be associated with brainstem dysfunction which is clinically characterized by a heterogeneous abolition of brainstem reflexes (56), impaired heart rate variability with a decrease in the sympathovagal balance (57), baroreflex and respirate rate variability, and also increased latencies of the somatosensory and middle-brain auditory evoked potentials (58) and absence of electroencephalographic reactivity (59). This brainstem dysfunction is associated with increased mortality, multiple organ failure, and disorders of consciousness, namely, coma and delirium (56). These phenomena are related to dysfunction of the reticular ascending activating system and the autonomic centers, which are both liable to neuro-inflammatory insult as evidenced by neuropathological studies (60). Septic patients often complain of anxiety, which is considered a warning signal. In a prospective, multicenter cohort study, we found that anxiety was more intense in patients who would subsequently develop new organ failure. It is conceivable that anxiety is either a marker of critical illness severity or, by notably increasing the allostatic load secondary to an overstimulation of the sympathetic system, an aggravating factor (61). Interestingly, we found that impaired perception of danger was also predictive of organ failure, suggesting that a dysfunction of the limbic system may contribute to unfavorable outcome (personal data). We conducted an experimental study to assess whether sepsis is associated with amygdala dysfunction. We found that there is an acute activation of the central amygdala-BNST circuits, whose specific inhibition prevents anxiety-related behaviors and fear memory 15 days after sepsis in mice (62).

The dysfunction of the autonomic or limbic centers is likely to result from a dysregulated neuro-inflammatory process, involving a neurotoxic activation of microglial cells. Microglial activation in response to a stimulus encompasses morphological, immunological, and metabolic changes. Activated microglial cells can, very schematically, acquire either a pro-inflammatory or an anti-inflammatory immunophenotype which are usually considered to be neurotoxic or neuroprotective, respectively. Activated microglia can release various neurotoxic mediators—namely, cytokines, NO, gliotransmitters and metabolites (such as reactive oxygen species)—that increase neuronal excitability, fuel neuronal hyper-activation, and may induce neuronal apoptosis (63, 64). The modulation of microglial activity might therefore be a relevant approach for restoring an adaptive immune-brain crosstalk in sepsis. There have been promising results from numerous experimental studies which have tested therapeutic interventions, namely, minocycline (65), hydrocortisone (66), cholinergic inhibition (67, 68), and vagal nerve stimulation (69). However, no randomized clinical trial has so far been successful. Administration of rivastigmine (70) has been shown to be deleterious and statins inefficient for treating or preventing brain dysfunction in critically ill patients (71, 72). Given that microglial activation is a dynamic phenomenon, a major limitation is the absence of a biomarker for the microglial phenotype. Although shown to be useful in patients with Alzheimer’s disease, positron emission tomography of microglial activation cannot be performed easily in septic patients (73). Based on experimental evidence of its effect on microglial cell-mediated neurotoxicity, we will soon carry out a multicenter, randomized clinical trial for assessing whether levetiracetam, an anti-seizure drug, prevents brain dysfunction in septic patients (73). The autonomic modulation of the immune response has also been investigated (74). It mainly consists of stimulation of the cholinergic anti-inflammatory reflex, which can be achieved by electrical stimulation of the vagal nerve, administration of agonists of nicotinic acetylcholine or β2-adrenergic receptors, but also by pharmacologic inhibition of cholinesterase (67) and electroacupuncture (75).

## The Brain-Immune Crosstalk in Ischemic Stroke

There are several arguments for hypothesizing that ischemic stroke can impair the brain-immune crosstalk. First, the latter can be activated by the systemic inflammatory response transitorily triggered by an ischemic stroke (76) but also by an infection, which is a frequent stroke complication favored by secondary peripheral immunodepression (77). Second, ischemic stroke can damage brain circuits involved in the immune-brain crosstalk, resulting in a maladaptive response to stress, with acute and long-term consequences. Finally, the activation of the immune system during sepsis can induce ischemic stroke.

Due to blood–brain barrier breakdown, the brain ischemic tissue releases cytokines, chemokines, brain-derived antigens, and DAMPS into the circulation, which stimulates the peripheral immune system. The brain-derived antigens, mainly enolase, S100b and GFAP, are drained into the lymphatic system and the spleen through the CSF and serum (78), where they can activate macrophages and B and T cells. It has been experimentally shown that there is a dramatic production of pro-inflammatory cytokines by activated T cells of the lymph nodes after ischemic stroke (79, 80). In stroke patients, circulating antibodies against the N-methyl-D-aspartic acid receptor or myelin basic protein are detectable, and brain antigens are also detected in tonsils and lymph nodes (81). With the use of a labeling technique, it has been experimentally shown that spleen immune cells are found in the brain 24 to 96 h after stroke (82). These findings indicate that stroke induces autoreactive T cells, which have been reported to worsen brain injury in animals but are also considered to favor post-stroke autoimmunity. If not reported after stroke, auto-immune encephalitis can occur after a herpes simplex encephalitis (83). The main consequence of this systemic inflammatory response is the infiltration (84)—facilitated by the increased expression of adhesion molecules—of the brain by activated cells of innate (i.e., macrophages, neutrophils, dendritic and natural killer cells) and adaptive (i.e., T cells) immunity, inducing blood–brain barrier dysfunction *via* protease secretion, brain edema, the neuro-inflammatory process, and finally, brain damage. If innate immunity cells are usually considered deleterious, it seems however that T cells are more equivocal. For instance, γδ T cells, which reside primarily in the gut, can reach the ischemic brain *via* the meninges and contribute to microglial neurotoxic activation and neutrophil recruitment by secreting IL-17 (85). On the other hand, T cells have also been shown to promote neurogenesis, repair and remodelling (4, 86–88). Cellular DAMPs mainly include adenosine triphosphate (ATP), S100b, High-Mobility Group-Box-1 and peroxiredoxins; extracellular DAMPS include fibronectin, tenascin C, heparan sulphate, and hyaluronan (78). They are major players in local neurotoxic phenomena but also contribute to the activation of the peripheral immune system. Within the first hours following stroke there is an increase in circulating pro-inflammatory cytokines (i.e., IL-6 and TNF-α), which are mostly produced from peripheral immune cells (89, 90). Thrombolysis can be complicated by a systemic inflammatory response syndrome that is associated with poor outcome (91). Interestingly, the targeting of DAMPs, immune-signaling molecules (such as cytokines), microglial polarizations, macrophages, and T cells are now considered to be promising therapeutic strategies (78, 92).

This acute pro-inflammatory response is rapidly followed by immunodepression, which is teleologically considered to dampen brain infiltration by activated immune cells and to have a neuroprotective effect. This immunodepression, characterized by lymphopenia, deactivation of monocytes, depletion of splenic T-cells and natural killer cells, reduces splenic size (80), and also decreases the production of proinflammatory cytokines such as lymphocytic IFN-γ and monocytic TNF-α (78, 93), which are necessary for defense against bacterial infection. The main consequences of this immunodepression is infection, to which changes in gut microbiota might contribute by increasing plasma trimethylamine N-oxide levels (94, 95). Gut dysbiosis is also a determinant of post-stroke outcomes (96).

The key role of the spleen in the immune response (36), and gut microbiota in infection and recovery, highlights the implication of the HPA axis and autonomic nervous system, both components of brain-immune communication and, as discussed above, the response to stress and sickness behavior. First, there is an increase of plasma cortisol and catecholamine levels after stroke (4, 97). Second, the spleen is innervated by the noradrenergic neurons of the splenic nerve, which is regulated by the cholinergic neurons of the vagal nerve. This innervation is relatively anti-inflammatory, *via* the β2-adrenergic pathway (98). There is no direct cholinergic innervation of the spleen. In contrast to that in rodents, there is direct sympathetic innervation of the spleen in humans, which preferentially interacts with leukocytes and is potentially pro-inflammatory (50). Interestingly, it has been shown that sepsis can impair this direct sympathetic nerve (50). In addition, circulating catecholamines modulate the response of the splenic immune cells. Therefore, there is a subtle sympathetic/parasympathetic balance in the spleen immune response. Regarding the gastrointestinal system, the sympathetic nervous system may be deleterious, by disrupting production of intestinal mucin, gut permeability (*via* noradrenergic-mediated expression of TREM1), and composition of intestinal microbiota (78, 99), while the vagal nerve is, on the other hand, more protective. For instance, reducing parasympathetic nerve activity after an acute brain injury stimulates intestinal bacterial proliferation and increases bacterial translocation (96). It has been recently demonstrated that there are circulating molecular regulators, such as small RNA, that finely control the cholinergic reflex in stroke patients. Therefore, targeting the autonomic nervous system seems a relevant therapeutic option. The blockade of spleen noradrenergic control was beneficial in an animal model of ischemic stroke with improvement in survival and prevention of infection (79, 100). Moreover, activating the cholinergic reflex reduces systemic and neuro-inflammation but also infarct size in stroke animal models (101, 102). The decrease in plasma acetylcholinesterase levels, a marker of cholinergic immunosuppressive activity and predictor of poor outcomes in stroke patients (103), supports intervention in the parasympathetic system. However, we call for caution as the sympathetic/parasympathetic balance is dynamic and complex, suggesting that its control cannot be simplistic. Among other benefits, such as replacing brain dead cells and promoting brain repair, stem cells might be a promising way to optimize neuro-immune interaction (104). We would like to emphasize that aging, a major risk factor for stroke, has a dramatic impact on peripheral and brain immune cells by favoring a pro-inflammatory response, but it also affects the HPA axis and autonomic nervous system, therefore altering the brain-immune crosstalk (78). Finally, it would be interesting to assess how sickness behavior is impaired in stroke patients, especially in those who developed infection or late anxiety and depression (105, 106). Furthermore, it remains unknown to what extent stroke location alters the peripheral-central crosstalk at play in the natural course of stroke. Notably, brainstem stroke may impair the connectivity between the autonomic, neuroendocrine, and limbic systems. Thus, if sepsis is a consequence of stroke-induced immunosuppression, it is also a factor in dysregulated neuro-immune responses and unfavorable outcomes in stroke patients.

To date, no intervention has been proven to decrease the risk of subsequent infection after stroke, and no study on the modulation of inflammation has been shown to prevent the occurrence of an ischemic stroke or to improve the outcome. For instance, the STROKE-INF clinical trial did not show any benefit for prophylactic antibiotics in reducing the risk of pneumonia in stroke patients with dysphagia (107). A better understanding of the brain-immune crosstalk could help develop a targeted and personalized therapeutic approach based on immune response phenotyping.

Finally, the systemic inflammatory response is associated with endothelial activation and intravascular coagulation, both of which can induce ischemic stroke, as reported in neuroradiological and neuropathological studies (108, 109). In addition to microcirculatory impairment, a decrease in blood pressure or cerebral blood flow, and impaired cerebral autoregulation, are also mechanisms of cerebral infarcts. Therefore, the occurrence of sepsis can worsen ischemic damage in a stroke patient, by triggering neuro-inflammation but also by affecting cerebral perfusion by inducing macro- and microcirculatory dysfunction (110).

## Concluding Remarks

In conclusion, brain-immune communication mainly involves the autonomic nervous system, which can sense and modulate peripheral inflammation, in cooperation with the neuroendocrine and limbic systems. Brain-immune crosstalk is a key player in the evolution of sepsis and stroke, both at its acute and recovery phase, influencing neurotoxic and neuroprotective mechanisms. It seems that the sympathetic nervous system is somewhat deleterious while the parasympathetic system tends to be beneficial. Targeting the autonomic nervous system would therefore be relevant but tremendously challenging since the sympathetic and parasympathetic activities are dynamically balanced and not univocal. Moreover, the natural neuro-immune response to stroke is likely to be modified by various but common factors, namely, aging and sex, and also stroke location and occurrence of sepsis.

## Author Contributions

All authors listed have made a substantial, direct, and intellectual contribution to the work and approved it for publication.

## Conflict of Interest

The authors declare that the research was conducted in the absence of any commercial or financial relationships that could be construed as a potential conflict of interest.

## Publisher’s Note

All claims expressed in this article are solely those of the authors and do not necessarily represent those of their affiliated organizations, or those of the publisher, the editors and the reviewers. Any product that may be evaluated in this article, or claim that may be made by its manufacturer, is not guaranteed or endorsed by the publisher.
